# Exploring potential network pharmacology-and molecular docking-based mechanism of melittin in treating rheumatoid arthritis

**DOI:** 10.1097/MD.0000000000034728

**Published:** 2023-08-11

**Authors:** Linfu Yang, Wenzheng Zhao, Xueyang Gong, Dan Yue, Yiqiu Liu, Yakai Tian, Kun Dong

**Affiliations:** a Yunnan Provincial Engineering and Research Center for Sustainable Utilization of Honey Bee Resources, Eastern Bee Research Institute, College of Animal Science and Technology, Yunnan Agricultural University, Kunming, China.

**Keywords:** melittin, molecular docking, network pharmacology, potential mechanism, rheumatoid arthritis

## Abstract

Rheumatoid arthritis (RA) is a type of difficult-to-cure arthralgia with a worldwide prevalence. It severely affects people’s living standards. For a long time, bee venom has been used to treat RA and has shown good results. Melittin is the main active component of bee venom used for RA treatment, but the molecular mechanism of melittin in RA treatments remains unclear.

**Methods:**

Potential melittin and RA targets were obtained from relevant databases, and common targets of melittin and RA were screened. The STRING database was used to build the PPI network and screen the core targets after visualization. The core targets were enriched by Gene Ontology functional annotation and Kyoto Encyclopedia of Genes and Genomes pathway. Finally, the binding of melittin to target proteins was evaluated through simulated molecular docking, which verified the reliability of the prediction results of network pharmacology.

**Results:**

In total, 138 melittin targets and 5795 RA targets were obtained from relevant databases, and 90 common targets were obtained through intersection. Eighteen core targets, such as STAT3, AKT1, tumor necrosis factor, and JUN, were screened out. Enrichment analysis results suggested that melittin plays an anti-RA role mainly through tumor necrosis factor, interleukin-17, toll-like receptors, and advanced glycation end products–RAGE signaling pathways, and pathogenic bacterial infection. Molecular docking results suggested that melittin has good docking activity with core target proteins.

**Conclusion:**

RA treatment with melittin is the result of a multi-target and multi-pathway interaction. This study offers a theoretical basis and scientific evidence for further exploring melittin in RA therapy.

## 1. Introduction

Rheumatoid arthritis (RA) is a systemic, chronic, and autoimmune disease of unknown etiology. RA onset is accompanied by substantial pain, mainly manifesting as joint injury, bone erosion, and edema of surrounding tissues. If uncontrolled, RA leads to joint stiffness, deformity, limited movement, and permanent loss of function.^[1]^ Synovial inflammation is a crucial pathological characteristic of RA, accompanied by obvious pannus formation, histiocytic proliferation, inflammatory cell infiltration, cell surface adhesion molecules, and changes in protease and cytokine expression levels.^[2]^ RA mainly occurs in middle-aged people, affecting at least more than 20 million people worldwide annually. The RA prevalence rate in some developed countries has reached 0.5% to 1%, and the prevalence rate in women is approximately 2.5 times of that in men.^[3]^ RA can cause other complications, such as cardiovascular and neurological diseases, and accordingly, the expected survival period of RA patients may be reduced by 3 to 10 years.^[4]^ The prevalence and incidence of RA are gradually increasing, which poses a serious threat to the human health and living standards.

The drugs currently used for RA treatment at clinics mainly include nonsteroidal anti-inflammatory drugs, glucocorticoids, disease-modifying antirheumatic drugs, and biological agents. These drugs mainly inhibit immune factors and inflammatory mediators to slow disease progression.^[5,6]^ They have their own advantages and disadvantages in RA treatment, but most drugs produce significant therapeutic effects only when used at a high dose and for a long term. Many of these drugs have serious side effects, including hearing loss, gastrointestinal damage, kidney irritation, and increased risk of cardiovascular diseases.^[7,8]^ Some studies have reported that better therapeutic effects can be achieved by combining drugs or using new drugs such as biologics. However, whether used in combination or alone, the therapeutic effect and side effects of drugs are closely related to their dosage.^[9]^ In addition, biologics are expensive and associated with risks; therefore, using them in large quantities in the clinic is difficult.^[10]^ Therefore, exploring potential therapeutic drugs for RA with significant efficacy and high safety is very necessary.

Bee venom is a transparent liquid secreted by the poison gland of bees and discharged by the tail stinger. Bee venom has long been used to treat various human diseases, such as some inflammatory and central nervous system diseases, and has anticancer potential.^[11,12]^ Recent studies have demonstrated a good outcome with the use of bee venom in the treatment of arthritis; this treatment approach has become popular in several countries and regions.^[13,14]^ Melittin is the main ingredient of bee venom, which accounts for approximately 40% to 60% of the dry weight of bee venom. This main active component of bee venom has been proven to play its role. Melittin is composed of 26 amino acid residues and has anti-inflammatory and pathogenic bacterium- and virus-killing effects.^[11,15]^ The main action mechanism of melittin against inflammation has been inhibition of NEMO, CD14, toll-like receptors 2 (TLR2), TLR4, and PDGFRβ signaling pathways, thereby reducing the activation of AKT, p38, PLCγ1, and ERK1/2 and nuclear translocation of nuclear factor kappa-B (NF-κB). Ultimately, the production of interleukin (IL)-1β, IL-6, tumor necrosis factor (TNF)-α, NO, and PGE2 pro-inflammatory factors is reduced, which thus reduces inflammation of the skin, joints, aorta, liver, and nerve tissue.^[16]^ Clinical studies have shown that melittin can inhibit NF-κB p65 and STAT3 activation as well as the expression of antiapoptotic genes such as mitochondrial cytochrome c and Bcl-2, significantly damage the activity of fibroblast-like synoviocytes in the joints of RA patients, promote fibroblast-like synoviocytes apoptosis and autophagy, reduce IL-1β and TNF-α production, effectively prevent RA-induced joint damage, and slow down the arthritis process.^[17]^ Therefore, melittin is a promising treatment option for RA. However, research on the action mechanism of melittin in RA treatment is insufficient, and little is known about the underlying molecular mechanism of its action.

Network pharmacology is a new method for studying drugs systematically and comprehensively. It combines the ideas of multidirectional pharmacology and systems biology. Constructing a complex network model of “drug–target–disease” would help analyze the possible action mechanism of drugs in treating diseases, and thus, pharmacological research can be transformed from the traditional search for a single target to a more comprehensive network analysis.^[18]^ More importantly, researchers cannot directly conduct experiments on humans. However, network pharmacology can integrate information from the human body to match databases of drugs and diseases. By mapping protein–protein interaction (PPI) networks, simulating molecular docking and so on, it can mimic drug use in humans and predict the targets that play a therapeutic role. Scientists have confirmed that the main component of bee venom, melittin, plays a critical role in the anti-inflammatory effect of bee venom, but the specific action mechanism of its treatment of arthritis remains clear. Thus, this study purposes to clarify the specific action mechanism of melittin against RA by using network pharmacological methods and verify the results through simulated molecular docking, so as to offer a new basis and ideas for basic research and related clinical applications.

## 2. Materials and methods

### 2.1. Collection and screening of melittin targets

PubChem (https://pubchem.ncbi.nlm.nih.gov) is a communal repository for information on compounds and their biological activities, making it an important source of chemical information for biomedical researches.^[19]^ The two-dimensional (2D) structure and target information of melittin (PubChem CID: 16133648) was downloaded in the SDF format from the PubChem chemical database. The 2D structure of melittin was uploaded to a drug and target analysis website (http://lilab-ecust.cn/pharmmapper),^[20,21]^ followed by downloading the target information of melittin. The gene names associated with the target proteins were then converted through the UniProt (http://www.uniprot.org) database, and only the human genes were screened.^[22]^

### 2.2. Collection and screening of RA targets

DisGeNET (https://www.disgenet.org) is used to store information about genes and variants related to human diseases^[23]^; GeneCards (https://www.genecards.org) is a systemic integration of human genetic information platform^[24]^; Online Mendelian Inheritance in Man (OMIM, https://www.omim.org) collects data on human genes and genetic diseases.^[25]^ Search for disease keyword “Rheumatoid Arthritis” was made in the DisGeNET, GeneCards, and OMIM databases to download the RA target information.

### 2.3. Identify common targets, constructing PPI network, and determining the core targets

Venny (version 2.1.0, https://bioinfogp.cnb.csic.es/tools/venny) was used to draw the Venn diagram for demonstrating the common targets of melittin and RA. The known and predicted protein–protein interactions were investigated with reference to the STRING database (https://string-db.org).^[26]^ The connection between common targets was delved using STRING with *Homo sapiens* as the selected species. A full network was built with a strong confidence level of 0.7, and the disconnected nodes were hidden. All other parameters were kept at the default settings. The visualization of the PPI network was performed using Cytoscape (version 3.9.1), and the CytoNCA (version 2.1.6) plug-in was used to obtain the basic data of common targets and its relationship data. The nodes with choice degree value > 2 times the median value of all network nodes and whose “betweenness centrality” and “closeness centrality” values were higher than the median value of all network nodes served as the core targets.^[27,28]^ They were then used to build a hub PPI network and analyze the interaction strength between the core targets.

### 2.4. Gene Ontology and Kyoto Encyclopedia of Genes and Genomes enrichment analyses

The purpose of enrichment analyses was to determine the biological pathways corresponding to the role of target genes. In this study, the core targets of melittin against RA were imported into DAVID (https://david.ncifcrf.gov) bioinformatics resource database,^[29]^ and the core targets were enriched by Gene Ontology (GO, http://www.geneontology.org) annotation and Kyoto Encyclopedia of Genes and Genomes (KEGG, https://www.kegg.jp/) pathway.^[30,31]^ Similarly, “Homo sapiens” was used as the species option, and *P* < .05 was set as the threshold for statistical significance. http://www.bioinformatics.com.cn is a composition and visual chart online platform that was used to map the top 10 enriched GO terms and the top 15 enriched pathways.

### 2.5. Molecular docking analysis

AutoDock is a large-range molecular simulation software that is mainly used to simulate ligand-protein molecular docking and examine the interaction of biological macromolecules and small molecule complex.^[32]^ In this study, the molecular docking of melittin-RA core target proteins was simulated by AutoDock to confirm their interaction, while the binding situation was evaluated to verify whether the prediction results of network pharmacology were reliable. The crystal structures of the target proteins were obtained from the RCSB Protein Data Bank (https://www.rcsb.org) and saved in the PDB format files; PyMOL (version 2.5.4) was used to eliminate the existing water molecules and small molecules. Subsequently, AutoDock Tools (version 1.5.7) was used to add hydrogen atoms to the target proteins and save them in the pdbqt format as receptor files, while melittin was saved in the pdbqt format as ligand files. Then, the grid box was set to cover the entire receptor protein. Finally, molecular docking simulations were conducted with AutoDock Vina (version 1.1.2) using the default parameters. The binding free energy was measured and the lowest energy was selected as the final docking conformation. The docking results were visualized by using Ligplot+ (vision 2.2.7) with two-dimensional map and PyMOL three-dimensional map.

## 3. Results

### 3.1. Obtaining melittin-related target

**Figure 1.**
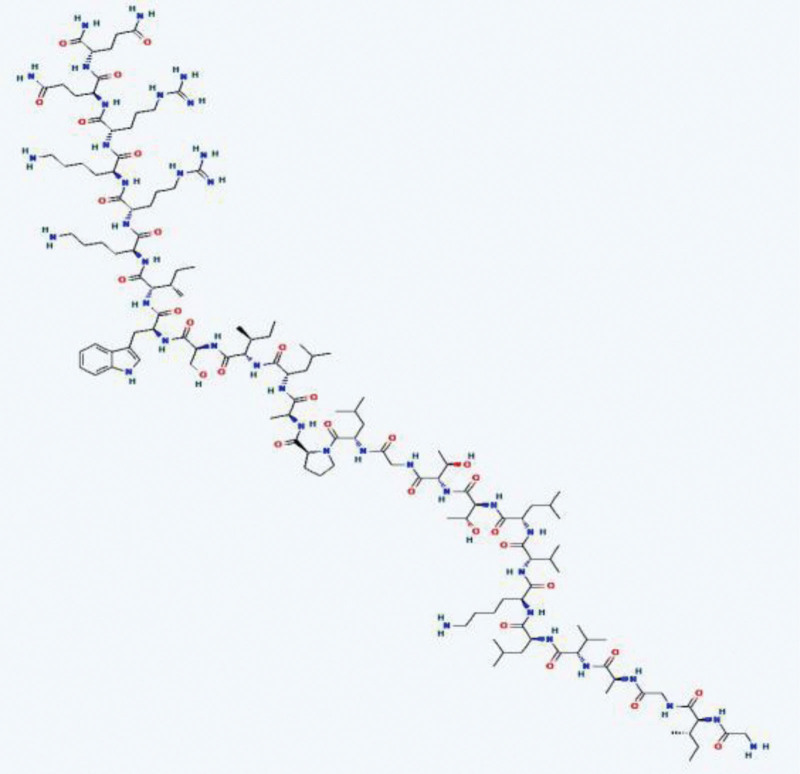
2D structure of melittin (download from the PubChem database, https://pubchem.ncbi.nlm.nih.gov).

The 2D structure of melittin (Fig. 1) and its 65 target genes were obtained from the PubChem database. The results, which were downloaded from the Drug and Target Analysis website (http://lilab-ecust.cn/pharmmapper), yielded 75 human-related melittin targets. Results from these 2 databases obtained 138 melittin-related targets by removing duplicate values.

### 3.2. Obtaining RA-related targets

In total, 2723 and 4956 RA-related targets were downloaded from the DisGeNET and GeneCards databases, respectively. The search results were downloaded from the OMIM database, and the non-genetic data were excluded to obtain 127 target genes. After removing duplicate values from the results obtained from the aforementioned 3 databases, 5795 RA-related targets remained.

### 3.3. Common targets of melittin and RA, PPI network, and core targets

**Figure 2.**
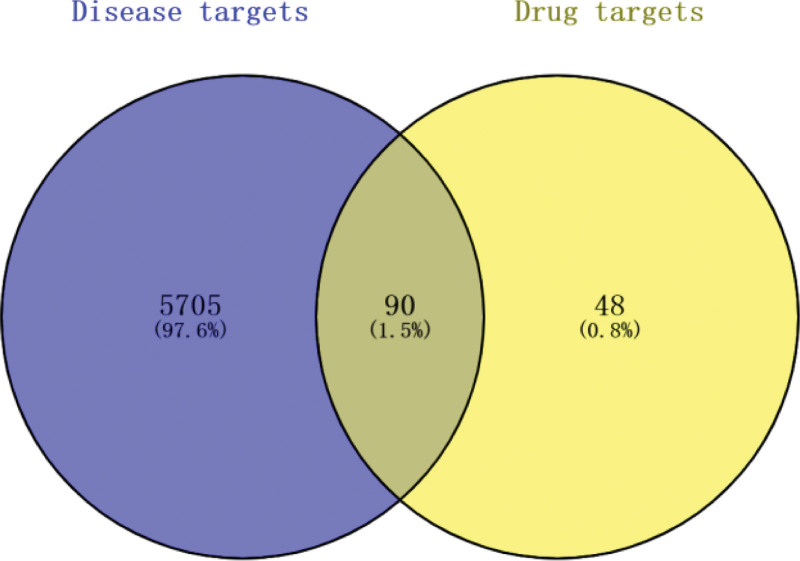
The Venn diagram of melittin and RA targets. The blue areas represent the potential targets for RA, the yellow areas represent the potential targets for melittin, and the gray areas represent their common targets. RA = rheumatoid arthritis.

**Figure 3.**
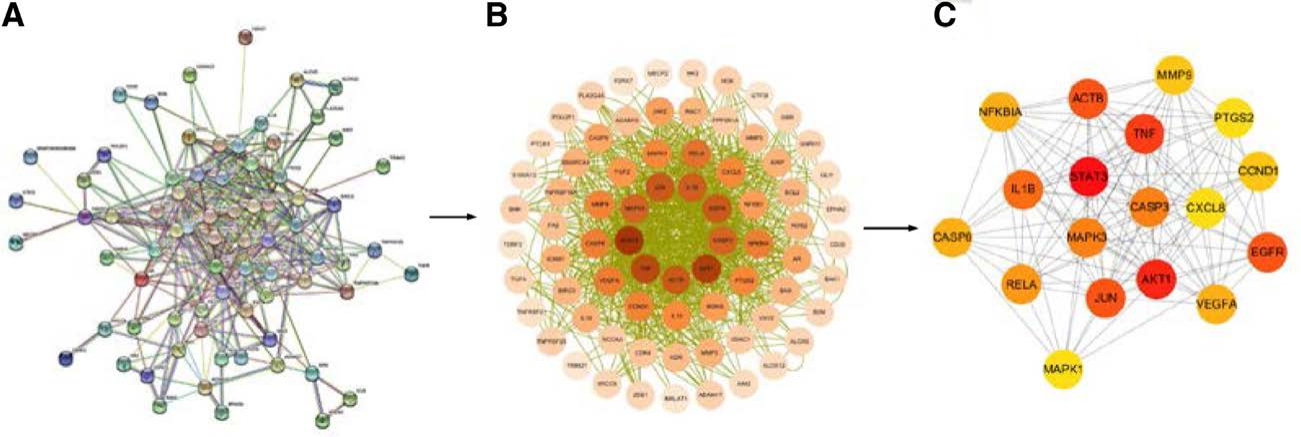
PPI network of the common targets of melittin-RA. (A) The original PPI network obtained in STRING. (B) The PPI network after visualization. (C) Hub PPI network. Nodes in (A–C) represent the protein targets. The color of nodes in (B) and (C) is proportional to the number of edges. The darker the color, more the numbers of edges, stronger the interaction between nodes, and greater the significance in the PPI network. PPI = protein–protein interaction, RA = rheumatoid arthritis.

Among 138 melittin-related targets and 5795 RA-related targets, 90 predicted targets of melittin against RA were obtained using Venny2.1.0 for intersection (Fig. 2). The STRING database was used to build PPI networks with these common targets. After clearing disconnected nodes, a PPI network diagram containing 74 nodes and 818 edges was obtained (Fig. 3A). However, the original PPI network was often crude. After visualization on Cytoscape, the obtained PPI network can more directly reflect the information of each node (Fig. 3B). Eighteen core targets were selected according to the set conditions: STAT3, AKT1, TNF, JUN, EGFR, ACTB, IL1B, CASP3, MAPK3, RELA, CASP8, VEGFA, NFKBIA, MMP9, CCND1, MAPK1, PTGS2, and CXCL8. Table 1 shows the basic information of the 18 core targets that together form the hub PPI network, consisting of 18 nodes and 122 edges (Fig. 3C).

**Table 1:** Information about the core targets in the hub PPI network.

Number	Targets name	Degree	Betweenness	Closeness
1	STAT3	70	586.11	0.63
2	AKT1	66	466.59	0.61
3	TNF	62	334.93	0.59
4	JUN	56	396.18	0.58
5	EGFR	56	748.58	0.58
6	ACTB	56	265.76	0.58
7	IL1B	54	299.23	0.55
8	CASP3	52	263.61	0.57
9	MAPK3	52	179.47	0.57
10	RELA	46	218.48	0.54
11	CASP8	42	186.16	0.52
12	VEGFA	42	57.92	0.53
13	NFKBIA	42	38.59	0.51
14	MMP9	40	35.47	0.53
15	CCND1	40	144.64	0.53
16	MAPK1	38	132.92	0.53
17	PTGS2	38	263.88	0.53
18	CXCL8	38	71.58	0.52

PPI = protein–protein interaction.

### 3.4. GO biological function and KEGG pathway enrichment analysis

#### 3.4.1. GO enrichment analysis.

**Figure 4.**
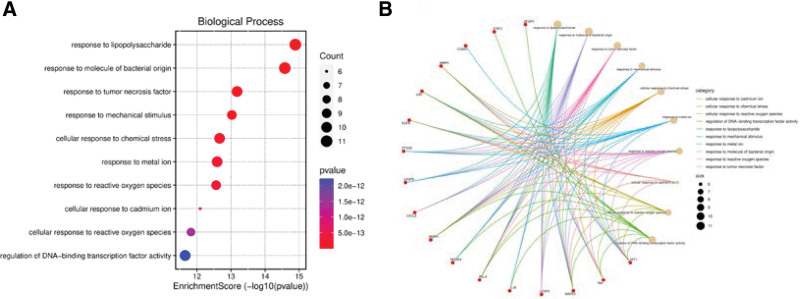
(A) Bubble chart of the pathway from GO-BP enrichment analysis. (B) The pathway-target relationship network of GO-BP enrichment analysis. BP = biological process, GO = Gene Ontology.

**Figure 5.**
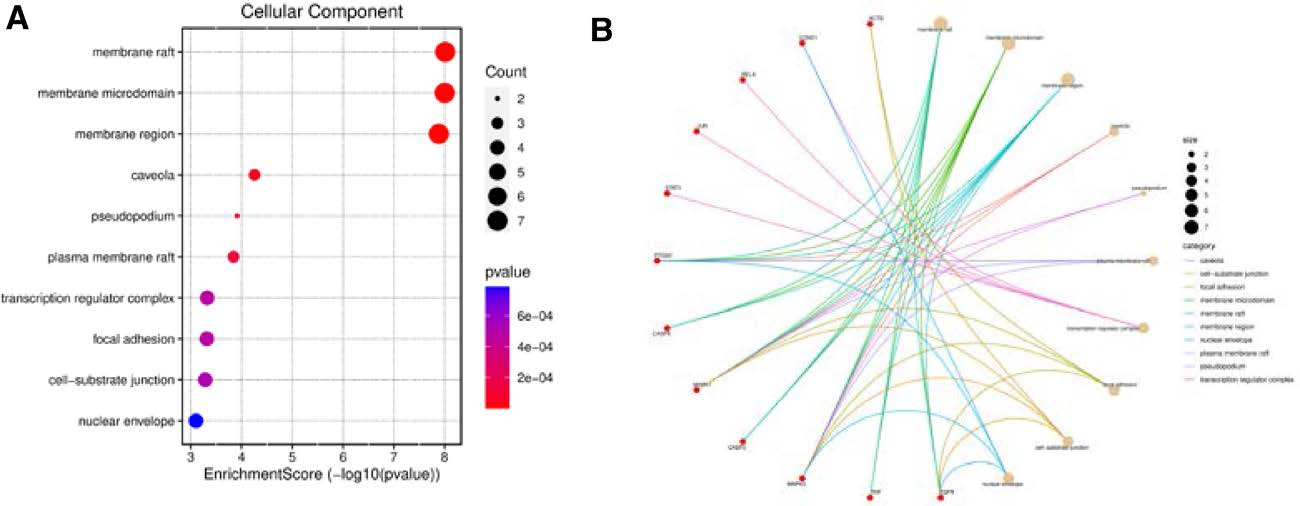
(A) Bubble chart of the pathway from GO-CC enrichment analysis. (B) The pathway-target relationship network of GO-CC enrichment analysis. CC = cell component, GO = Gene Ontology.

**Figure 6.**
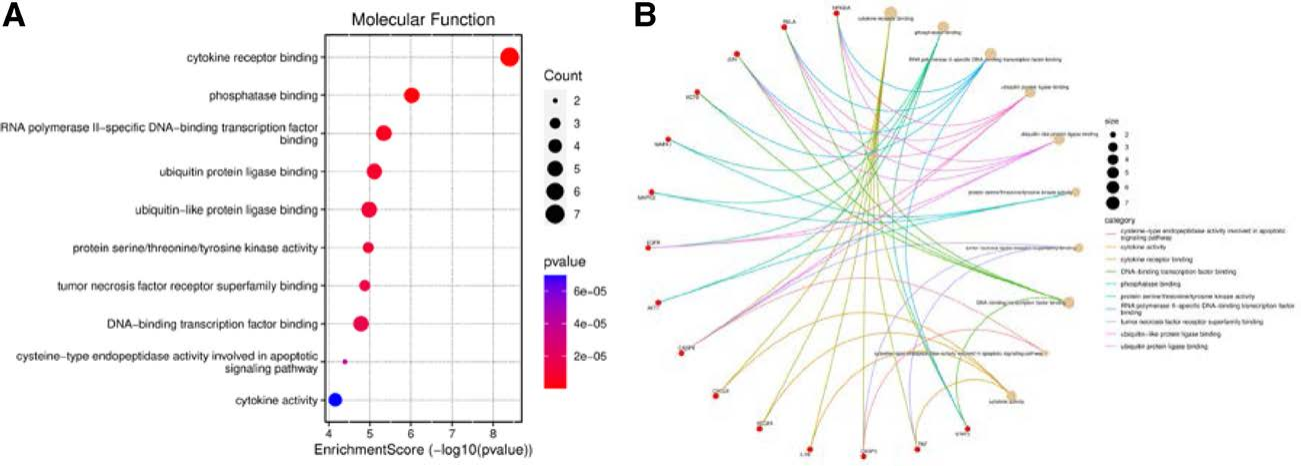
(A) Bubble chart of the pathway from GO-MF enrichment analysis. (B) The pathway-target relationship network of GO-MF enrichment analysis. MF = molecular function, GO = Gene Ontology.

Through GO functional annotation analysis, 1674 GO items were collected, including 1537 biological process items, 45 cell component items, and 92 molecular functional items, *P *< .05. The *P* value of each enrichment result was calculated and arranged in order from smallest to largest. Then, the enrichment scores of the top 10 items in each category of biological process, cell component, and molecular functional were counted, and the path–target relationship network was plotted (Figs. 4–6). GO functional enrichment analysis suggested that the predicted targets of melittin against RA were compactly related to the following biological processes: response to lipopolysaccharide (LPS), bacteria, TNF, metal ions, reactive oxygen species (ROS), response to mechanical stimulation and chemical stress, and regulation of DNA-binding transcription factor activity. They were closely related to the following cellular components: membrane region or membrane structure such as the membrane microdomain, membrane raft and nuclear membrane, pseudopodium, focal adhesion, transcriptional regulatory complex, and cell–substrate junction site. They were closely related to the following molecular functions: cytokine receptor binding, ubiquitin protein–ligase binding, phosphatase binding, and protein serine/threonine/tyrosine kinase activity.

#### 3.4.2. KEGG enrichment analysis.

**Figure 7.**
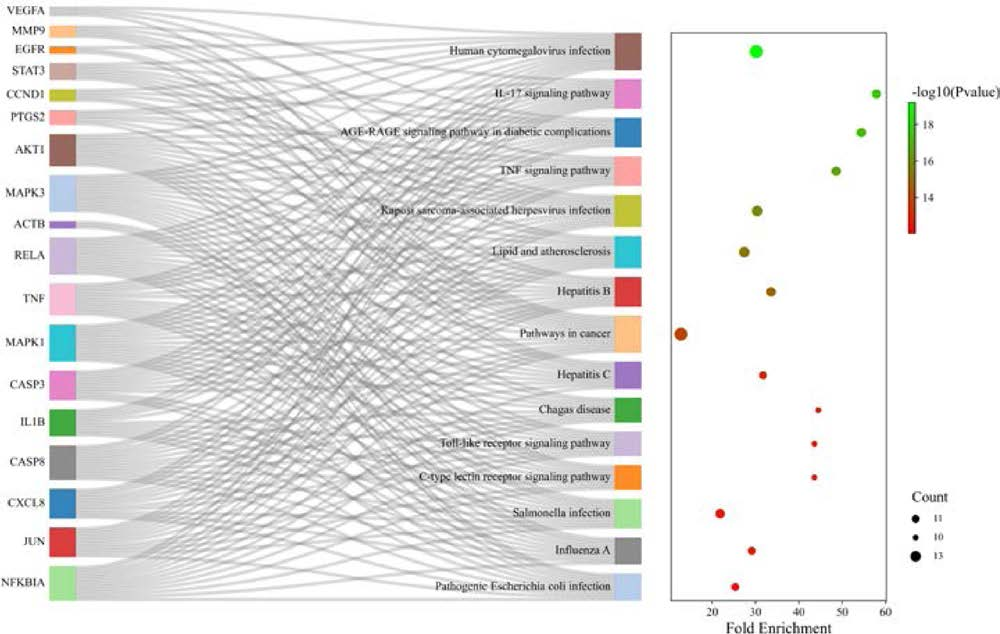
KEGG enrichment analysis of the top 15 pathways. The core target is on the left, and the pathway is on the right. The number of enriched core targets in each KEGG item is depicted as a circle, and *P* values were indicated in different colors. KEGG = Kyoto Encyclopedia of Genes and Genomes.

The KEGG pathway analysis suggested that 18 core targets were enriched in 139 related pathways. Figure 7 shows the first 15 pathways. Table 2 lists the top 15 pathways and detailed information about targets, and at least 10 core targets were enriched in each pathway. The main pathways were as follows: human cytomegalovirus infection, cancer, lipid and atherosclerosis, Kaposi sarcoma-associated herpesvirus infection, hepatitis B, hepatitis C, IL-17 signaling pathway, TNF signaling pathway, Salmonella infection, advanced glycation end products (AGE)–RAGE signaling pathway in diabetic complications, TLR signaling pathway, influenza A, pathogenic Escherichia coli infection, C-type lectin receptor signaling pathway, and Chagas disease. The aforementioned analysis implies that melittin may play a therapeutic role against RA by participating in various biological regulatory processes.

**Table 2:** Information about the top 15 KEGG pathways.

Description	Fold enrichment	*P* value	Gene ID	Count
Human cytomegalovirus infection	30.24	6.14E-20	CXCL8/STAT3/PTGS2/TNF/EGFR/RELA/VEGFA/NFKBIA/CASP8/CCND1/IL1B/CASP3/AKT1/MAPK1/MAPK3	15
Pathways in cancer	12.81	1.16E-14	MMP9/JUN/CXCL8/STAT3/PTGS2/VEGFA/NFKBIA/CASP8/CCND1/CASP3/AKT1/EGFR/RELA/MAPK1/MAPK3	15
Lipid and atherosclerosis	27.42	4.48E-16	JUN/CXCL8/STAT3/TNF/MMP9/RELA/NFKBIA/CASP8/IL1B/CASP3/AKT1/MAPK1/MAPK3	13
Kaposi sarcoma-associated herpesvirus infection	30.39	1.28E-16	JUN/CXCL8/STAT3/PTGS2/RELA/VEGFA/NFKBIA/CASP8/CCND1/CASP3/AKT1/MAPK1/MAPK3	13
TNF signaling pathway	48.60	2.26E-17	NFKBIA/IL1B/CASP3/JUN/PTGS2/TNF/RELA/MAPK3/CASP8/MAPK1/AKT1/MMP9	12
IL-17 signaling pathway	57.90	3.01E-18	NFKBIA/CASP3/MAPK1/JUN/MMP9/RELA/CXCL8/CASP8/IL1B/PTGS2/TNF/MAPK3	12
Salmonella infection	21.86	1.80E-13	CASP3/TNF/RELA/MAPK1/NFKBIA/JUN/CXCL8/CASP8/IL1B/AKT1/ACTB/MAPK3	12
Hepatitis B	33.60	1.48E-15	NFKBIA/RELA/CASP8/CASP3/STAT3/MAPK1/AKT1/JUN/CXCL8/TNF/MMP9/MAPK3	12
AGE-RAGE signaling pathway in diabetic complications	54.43	6.14E-18	JUN/AKT1/TNF/CXCL8/MAPK3/CCND1/IL1B/STAT3/MAPK1/RELA/VEGFA/CASP3	12
Pathogenic Escherichia coli infection	25.33	8.95E-13	NFKBIA/JUN/CXCL8/CASP8/IL1B/CASP3/MAPK1/TNF/RELA/ACTB/MAPK3	11
Influenza A	29.18	2.14E-13	NFKBIA/CASP3/MAPK1/CXCL8/CASP8/IL1B/AKT1/ACTB/MAPK3/TNF/RELA	11
Hepatitis C	31.78	8.99E-14	NFKBIA/MAPK1/AKT1/CASP8/CCND1/CASP3/STAT3/TNF/RELA/EGFR/MAPK3	11
Toll-like receptor signaling pathway	43.61	1.39E-13	NFKBIA/MAPK1/AKT1/JUN/CXCL8/CASP8/IL1B/MAPK3/TNF/RELA	10
C-type lectin receptor signaling pathway	43.61	1.39E-13	NFKBIA/JUN/TNF/CASP8/IL1B/MAPK1/PTGS2/RELA/MAPK3/AKT1	10
Chagas disease	44.47	1.16E-13	NFKBIA/JUN/MAPK1/AKT1/CXCL8/CASP8/IL1B/TNF/MAPK3/RELA	10

KEGG = Kyoto Encyclopedia of Genes and Genomes.

### 3.5. Molecular docking

**Figure 8.**
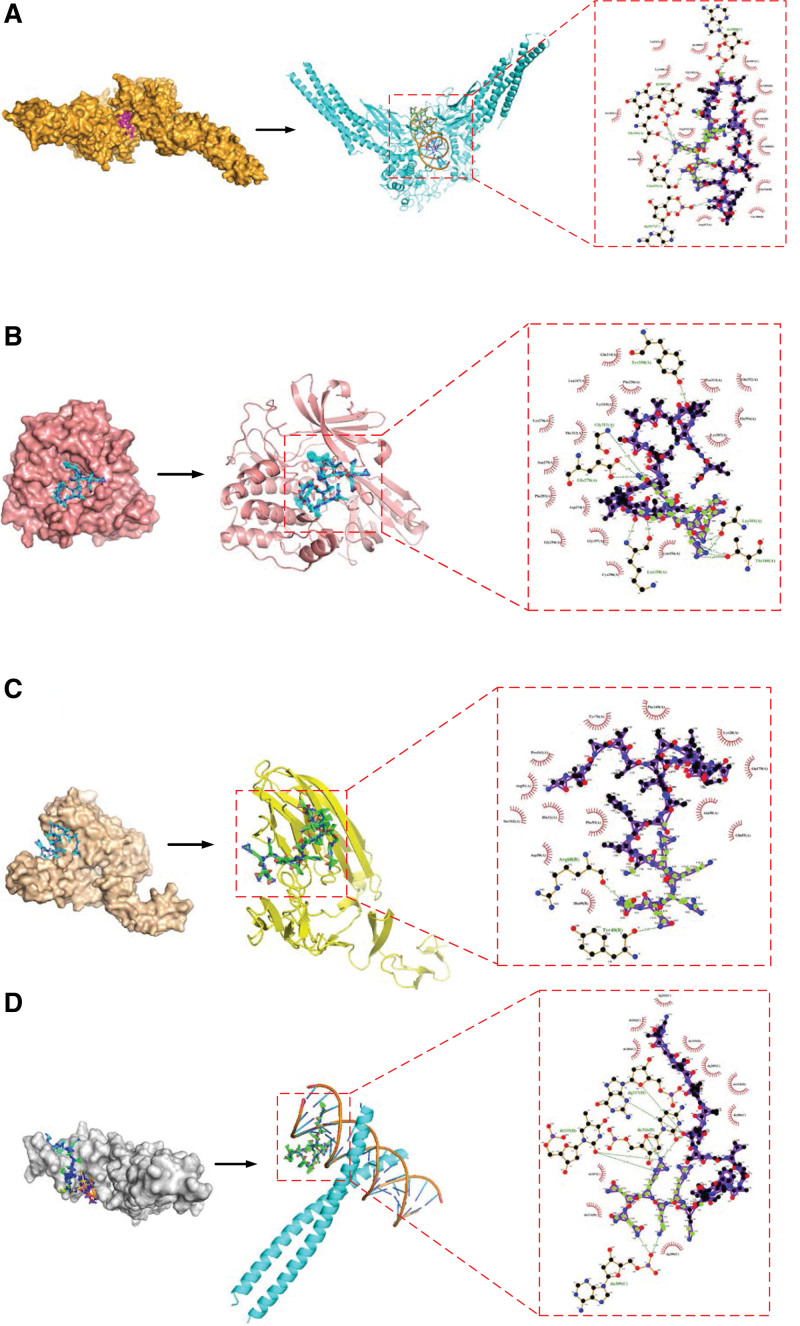
The docking model of melittin with the core target proteins (A) STAT3, (B) AKT1, (C) TNF, and (D) JUN (crystal structure model, spatial structure model, and 2D structure model). In the crystal structure and spatial structure models, the ligands are represented by colored stick, while the receptor proteins are represented by the crystal or banded substances. In the 2D structural model, the ligand is depicted as a purple stick, amino acid residues as a brown stick, hydrogen bonds as green dashed lines, and hydrophobic interactions as red stellations. TNF = tumor necrosis factor.

The 18 core target proteins were verified through molecular docking with melittin, and the results showed that the ligands had a favorable docking activity with the target proteins. The top 4 core target proteins (STAT3, AKT1, TNF, and JUN) were selected as representative examples to show their docking patterns (Table 3). In the docking mode between melittin and STAT3, melittin, as a ligand, formed 7 hydrogen bonds with different parts of the STAT3-encoded protein. Among which, the ligand formed 1 hydrogen bond each with Thr341, da1008, and dg1017 and 2 hydrogen bonds each with Glu415 and dt1007 (Fig. 8A). In the docking mode between melittin and AKT1, 11 hydrogen bonds were formed at different parts of the ligand and receptor. Among which, melittin formed 1 hydrogen bond each with Tyr350 and Gly311; 2 hydrogen bonds each with Lys158, Lys301, and Glu278; and 3 hydrogen bonds with Thr160 (Fig. 8B). The docking mode between melittin and TNF was relatively simple. In the docking results, the ligand formed 2 hydrogen bonds with the receptor, that is, melittin formed 1 hydrogen bond each with Tyr40 and Arg68 (Fig. 8C). The docking mode of melittin and JUN showed that the ligand and receptor formed 10 hydrogen bonds, in which melittin formed 2 hydrogen bonds each with da209, dt315 and dg317 and 4 hydrogen bonds with dc316 (Fig. 8D). In addition, abundant hydrophobic effects were observed in all the aforementioned docking modes.

**Table 3:** Molecular docking parameters and the energy changes of melittin and target proteins.

Targets name	PDB ID	Center_x	Center_y	Center_z	Size_x	Size_y	Size_z	Affinity (kcal/mol)
STAT3	6qhd	−44.32	−43.49	14.36	126	104	116	−5.6
AKT1	4ejn	32.07	45.23	15.2	58	66	70	−4.6
TNF	1tnr	41.52	23.62	47.22	86	94	104	−4.5
JUN	1jnm	10.201	0.493	31.336	64	110	126	−5.7

## 4. Discussion

As a cutting-edge approach, network pharmacology offers a comprehensive data platform for studying multidirectional pharmacology fusion systems biology. This method has been used in predictive studies of the “compound–protein/gene–disease” approach, which can systematically reveal the internal molecular mechanism underlying drug treatment of diseases.^[18]^ Therefore, network pharmacological method was used to predict the action mechanism of melittin against RA in this study, and a molecular docking study was conducted to estimate the reliability of the predicted targets.

In this study, 90 melittin anti-RA targets were predicted using relevant databases, accounting for 1.5% of the total targets. The PPI network was constructed, and 78 related targets remained after the disconnected nodes were removed. The results of the analysis suggested a strong interaction between these target genes. After further screening, 18 core targets were obtained, including STAT3, AKT1, TNF, JUN, IL1B, MAPK1/8, and VEGFA. Stronger interactions were observed between core targets, and importantly, most of these genes were associated with RA progression. For example, STAT3 is a key regulatory factor in RA occurrence and development. In 1 study, *STAT3* knockout or demethylation in RA mice reduced the differentiation frequency of immune cells and osteoclast activity, which hindered joint erosion.^[33,34]^ High levels of TNF-α and IL-1β, 2 common inflammatory cytokines, were detected in the synovial fluid of RA patients.^[35]^ Melittin was recently reported to inhibit IL-1β and TNF-α levels, and therefore, the activation of JNK (c-Jun N-terminal kinase) and P38MAPK decreased.^[16]^ Fibroblast expansion in the synovium of RA patients is the main cause of synovitis, and the expression of JUN family members can promote fibroblast proliferation and differentiation. JUN is a major component of AP-1, which determines the expression of many pro-inflammatory proteins in RA.^[36]^ MAPK protein kinase is involved in the signaling process from the cell membrane to nucleus and regulates cell growth, differentiation, stress, inflammation, and other cell pathological and physiological processes.^[37]^ Therefore, melittin may inhibit the activation of JUN and ERK1/2 (MAPK3, MAPK1) pathways, thereby effectively blocking inflammatory responses, which was confirmed in this study. Moreover, melittin reduces AKT activation and nuclear translocation of NF-κB.^[38]^ The PI3K/AKT pathway is closely related to the regulation of chondrocyte metabolism, and AKT1 activation promotes B cell proliferation and differentiation; these processes can accelerate RA deterioration.^[39]^ The nuclear factor NF-κB is among the crucial regulatory factors of proinflammatory gene expression. Melittin has been found to reduce IκB degradation by inhibiting IKK phosphorylation, such that more IκB in the cytoplasm can inhibit the nuclear translocation of 2 subunits of NF-κB (p65 and p50) and activate the transcription of inflammatory cytokines by related proinflammatory genes. It effectively alleviates RA progression.^[40]^

Second, GO functional annotation analysis provides a comprehensive source for gene function search. The GO analysis results showed that the potential targets of melittin for RA treatment are closely related to various pathways and components, such as the response to LPS, bacteria, TNF, and ROS, as well as the processes of cytokine–receptor binding, substrate dephosphorylation, and protein kinase activation. Important signal transduction involves cell structures such as the cell membrane and nucleus. These results are consistent with those of previous reports that they are key components or pathways of inflammation progression. LPS is a type of cellular endotoxin and is also the cell wall component of gram-negative bacteria. LPS has toxic effects on human or animal cells. LPS can activate TLR4 to stimulate the production of IL-6, IL-17, and TNF-α cytokines.^[41]^ LPS-binding protein easily binds to LPS, which is the first step in mediating an anti-inflammatory response, and LPS-binding protein can be used as a biomarker for RA diagnosis.^[42]^ CD14 is an LPS receptor, and melittin can inhibit the CD14 pathway and relieve inflammatory pathology.^[16]^ Studies have revealed that the gut microbiota composition in RA patients is different from that in the general population, and the level of some bacterial genera in the gut of RA patients is higher, which may interfere with the host’s relevant immune response.^[43]^ For example, the cell wall components of gram-positive bacteria and LPS also activate the P38MAPK pathway.^[44]^ Therefore, when used to treat RA, melittin may play a crucial role against pathogenic bacteria. Furthermore, insufficient oxygen supply to synovial immune cells in RA patients can upregulate the expression of hypoxia inducible factor-1α and lead to ROS production. A large amount of ROS causes serious damage to the cell structure and leads to oxidative stress.^[45]^ In 1 study, STAT3 was found to be a central pathway that regulates oxidative stress, inflammation, and apoptosis.^[46]^ These results suggest that melittin inhibits STAT3 expression and thus reduces ROS production, which may be one of the pathways for RA remission.

KEGG, developed by the University of Tokyo and Kyoto University in Japan, is a database for systematic analysis of gene function.^[31]^ In this study, by performing the KEGG analysis, the potential targets of melittin against of RA were associated with multiple signaling pathways, including TNF, IL-17, TLR, and AGE–RAGE signaling pathways, and bacterial and viral infections, which were mainly related to inflammatory diseases. TNF is a cytokine that can directly kill tumor cells and exhibits no obvious toxic effect on normal cells. It is mainly produced by activated macrophages. TNFR1 (p55) and TNFR2 (p75) are 2 TNF receptors, and TNF exerts its biological activity mainly by interacting with TNFR1. After binding with TNFR1, TNF activates NF-κB, c-Jun, and PI3K/Akt pathways. The expression of IL-1, IL-6 and GM-CSF cytokines is upregulated, as well as the production of NO and PGE2 is induced, which are considered as typical inflammatory factors.^[47]^ IL-17 is an inflammatory cytokine mainly produced by activated T cells, which promotes the inflammatory pathology of autoimmune diseases. Conversely, IL-17 also promotes T cell activation, activates the MAPK and NF-κB pathways, and stimulates the production of IL-6, IL-8, and TNF-a cytokines in macrophages and fibroblasts.^[48,49]^ In addition, IL-17 can effectively mediate neutrophil excitation, thus effectively mediating the inflammatory response of tissues.^[50]^ Meanwhile, TNF and IL-1 were also 2 important results when the core targets were screened for in this study. TLRs are a class of key factors involved in nonspecific immunity. They are activated to induce a strong immune response, which contributes to the body’s resistance to infections. However, excessive immune responses can lead to autoimmune diseases, such as the activation of most TLRs induces NF-κB and AP-1 pathways.^[51]^ Melittin inhibits TLR2 and TLR4 signaling pathways and is an effective treatment for RA.^[16]^ AGEs binding to its receptor RAGE can cause intracellular oxidative stress, lead to NF-κB activation, further stimulating the production of growth factors and cytokines. This process is also related to PKC, JAK-STAT, MAPK, and PI3K/Akt activation.^[52]^ Therefore, the AGE–RAGE signaling pathway also plays a vital role in inflammatory responses. Combined with previous reports, TNF, IL-17, and TLR signaling pathways are currently key pathways involved in the clinical treatment of RA.^[5,53,54]^ Importantly, these 3 pathways are included in the predicted results of this study, and so, we believe that other pathways predicted here have important reference value.

Molecular docking is a vital method for estimating whether the prediction results of network pharmacology are reliable. In this study, Autodock docking results revealed that most target proteins had certain binding activity. STAT3, AKT1, TNF, and JUN exhibited strong binding activities, suggesting that they bind tightly to melittin molecules. Moreover, the docking diagram showed that hydrogen bond accumulation and hydrophobic force are the main forms of interaction. To better analyze the stability and interaction between the drug and target, the binding free energy of the ligand–receptor interaction was also analyzed. Generally, the lower the energy when 2 substances undergo chemical reactions, the more stable the conformation of the complex, and interactions are more likely to occur under natural conditions.^[55]^ The results showed that all of the aforementioned binding free energies are less than zero, suggesting that the reaction could occur spontaneously, that is, melittin and the predicted targets could be better combined in the body. Thus, melittin has a good effect in RA treatment.

In summary, the study results clarified the internal molecular mechanism of action of melittin against of RA through network pharmacological means and verified the reliability of the predicted results through simulated molecular docking. However, this study has some limitations. Although we obtained melittin- and RA-related targets by referring to numerous databases, these data are certainly insufficient, because some of the latest research output are difficult to discover. Second, these targets and key pathways should be verified through additional experiments, and in vivo experiments of melittin against RA are required to explore effective medication methods and reasonable dosage. This would make it possible to put melittin into clinical and scientific use.

## 5. Conclusion

This study is the first to explore the potential action mechanism of melittin against RA by using network pharmacology and molecular docking methods. Data showed that melittin can treat RA through a multi-target and multi-pathway mechanism, and melittin’s therapeutic effect was believed to involve important hub targets such as STAT3, AKT1, TNF, JUN, IL1B, MAPK, and VEGFA. Melittin may play an anti-RA role through TNF, IL-17, TLR, and AGE–RAGE signaling pathways, and pathogenic bacterial infection. The study results provide a reference for further exploring the action mechanism of melittin in RA treatment. This valuable discovery offers theoretical support for the clinical application of melittin and the molecular design of therapeutic targets and pathways of RA.

## Author contributions

**Conceptualization:** Linfu Yang, Kun Dong, Yakai Tian.

**Data curation:** Linfu Yang, Dan Yue, Yiqiu Liu.

**Formal analysis:** Linfu Yang, Wenzheng Zhao, Xueyang Gong, Yakai Tian.

**Investigation:** Wenzheng Zhao, Xueyang Gong, Dan Yue, Yiqiu Liu.

**Methodology:** Kun Dong, Yakai Tian.

**Visualization:** Yakai Tian.

**Writing – original draft:** Linfu Yang.

**Writing – review & editing:** Kun Dong, Yakai Tian.
